# DNA as Therapeutics; an Update

**DOI:** 10.4103/0250-474X.58169

**Published:** 2009

**Authors:** P. Saraswat, R. R. Soni, A. Bhandari, B. P. Nagori

**Affiliations:** Mahatma Gandhi Medical College and Hospital, RIICO Institutional Area, Sitapura, Jaipur-302 022, India; 1 Jaipur Fertility and Microsurgery Research Center, Bani Park, Jaipur-302 016, India; 2 Department of Pharmacy, Jodhpur National University, Narnadi, Jhanwar Road, Jodhpur-342 001, India; 3 Department of Pharmaceutical Chemistry, L. M. College of Science and Technology, Shastri Nagar, Jodhpur-342 003, India

**Keywords:** Gene therapy, nucleic acid therapeutics, antisense, gene transfer technology, gene therapy trials, DNA delivery systems, viral vectors, nonviral vectors, liposomes

## Abstract

Human gene therapy is the introduction of new genetic material into the cells of an individual with the intention of producing a therapeutic benefit for the patient. Deoxyribonucleic acid and ribonucleic acid are used in gene therapy. Over time and with proper oversight, human gene therapy might become an effective weapon in modern medicine's arsenal to help fight diseases such as cancer, acquired immunodeficiency syndrome, diabetes, high blood pressure, coronary heart disease, peripheral vascular disease, neurodegenerative diseases, cystic fibrosis, hemophilia and other genetic disorders. Gene therapy trials in humans are of two types, somatic and germ line gene therapy. There are many ethical, social, and commercial issues raised by the prospects of treating patients whose consent is impossible to obtain. This review summarizes deoxyribonucleic acid-based therapeutics and gene transfer technologies for the diseases that are known to be genetic in origin. Deoxyribonucleic acid-based therapeutics includes plasmids, oligonucleotides for antisense and antigene applications, deoxyribonucleic acid aptamers and deoxyribonucleic acidzymes. This review also includes current status of gene therapy and recent developments in gene therapy research.

Human gene therapy is defined as the introduction of new genetic material into the cells of an individual with the intention of producing a therapeutic benefit[1–3]. A number of human diseases are known to be genetic in origin (Huntington's chorea and cystic fibrosis to name a few) and virtually all diseases, except for trauma, have a hereditary component[4]. Thus, the opportunity to treat such disorders by replacing the defective gene(s) with a normal healthy gene (gene therapy) offers a novel therapeutic approach for patients who suffer from such diseases. Now, gene therapy routinely is evoked to encompass the use of deoxyribonucleic acid (DNA) as a drug to alleviate the symptoms of a disease, even if the therapeutic genes are not strictly ‘corrective’ (in the sense of restoring a function known to be mutated in the affected cells). In its broadest terms, gene therapy represents an opportunity for the treatment of genetic disorders in adults and children by genetic modification of human body cells[5]. All of the gene therapy trials currently approved for use in human patients target somatic cells that will live only as long as the patient. This ensures that the genetic treatment will affect only one generation and will not alter the genetic makeup of any offspring of the patient, since there is no spread of the therapeutic gene(s) to the gametes. This is known as the somatic gene therapy, and its purpose is to alleviate disease in the treated individual alone. More than 300 clinical trials involving gene transfer in patients have been approved and the first nucleic acid drug, an antisense oligonucleotide, fomivirsen (marketed as Vitravene) has been approved by the United States Food and Drug Administration (USFDA) for the treatment of cytomegalovirus retinitis in immunocompromised patients[6]. In contrast, it is also possible to target directly the gametes (sperm and ova) in order to modify the genetic profile, not of the current, but of the subsequent generation of unborn ‘patients’. Gene transfer at an early stage of embryonic development also might have similar effects by achieving gene transfer to both somatic and germ line cells. This is known as the germ line gene therapy.

Apart from the inability to predict the long-term effects of altering the germ line by delivery of exogenous genetic material at the scientific level, there are many ethical, social, and commercial issues surround the technique. The social implications of such technology include the possibility that patients might suffer from depression as a result of being ‘genetically altered’ or might not be accepted by society in the way that they were before treatment. The commercial implications of such technology are that the insurance companies and other such institutions also would want to access the available information prior to them granting life insurance policies. So, it is obvious that a person shown to have a predisposition to a genetic disease could be severely penalized because of a mutation in their DNA, even though they might never develop the disease. The biggest setback for gene therapy occurred in 1999 when Jesse Gelsinger, an 18 year-old high-school graduate from Arizona, died as a result of a gene therapy experiment. Gelsinger developed a fever and blood clots throughout his body within hours of treatment to correct partial ornithine transcarbamylase (OTC) deficiency, a rare metabolic disease that can cause a dangerous build-up of ammonia in the body and died four days later[7]. Despite this attempt to preserve public confidence in gene therapy, the Washington post documented six unreported deaths on 3^rd^ November 1999 that had occurred in trials conducted at the Cornell Medical Center, Manhattan and at the Tufts University, Boston[8].

Nucleic acids are one of the most important sources not only for the understanding of the fundamental basis of human life but also for the development of a novel group of therapeutics. One of the significant advantages of DNA-based drugs over currently available low molecular weight pharmaceuticals is their selective recognition of molecular targets and pathways, which imparts tremendous specificity of action. DNA-based therapeutics includes plasmids, oligonucleotides for antisense and antigene applications[9], DNA aptamers, and DNAzymes. In gene therapy, the gene transfer technologies are DNA delivery systems for nucleic acid based therapeutics.

Although most of the DNA and RNA based drugs are in early stages of clinical trials, these classes of compounds have emerged in recent years to yield extremely promising candidates for drug therapy for wide range of diseases, including cancer, AIDS, neurological disorders such as Parkinson's disease and Alzheimer's disease, and cardiovascular disorders[10,11]. Elucidation of the human genome has also provided a major impetus in identifying human genes implicated in diseases, which may eventually lead to the development of DNA and RNA based drugs for gene replacement or potential targets for gene ablation[12]. The Human Genome project will help determine genetic markers responsible for patient response to drug therapy, drug interactions, and potential side effects[13]. Developments in human genomics, transcriptomics, and proteomics will provide an additional impetus for the advancement of DNA based therapeutics by supplying novel targets for drug design, screening, and selection. In this review, we summarize DNA-based therapeutics, gene transfer technologies, current status of gene therapy and recent developments in gene therapy research.

## DNA- BASED THERAPEUTICS

### Plasmids:

Plasmids are high molecular weight, double stranded DNA constructs containing transgenes, which encode specific proteins. On the molecular level, plasmid DNA molecules can be considered pro-drugs that upon cellular internalization employ the DNA transcription and translation apparatus in the cell to biosynthesize the therapeutic entity, the protein[14]. The mechanism of action of plasmid DNA requires that the plasmid molecules gain access into the nucleus after entering the cytoplasm. Nuclear access or lack thereof eventually controls the efficiency of gene expression. In addition to disease treatment, plasmids can be used as DNA vaccines for genetic immunization[15]. In the early stages of development, plasmid-based gene therapy was attempted to correct inheritable disorders resulting from a single gene defect. The first federally approved human gene therapy protocol was initiated in 1990 for the treatment of adenosine deaminase deficiency[16]. Since then, more than 500 gene therapy protocols have been approved or implemented[17]. In 2002, scientists reported the successful gene-therapy-based cure for severe combined immunodeficiency (SCID)[18]. In 2003, the Chinese drug regulatory agency approved the first gene therapy product for head and neck squamous carcinoma under the trade name Gendicine[19]. Currently, diseases with complex etiologies such as cancer[20–21] and neurodegenerative disorders such as Alzheimer's disease and Parkinson's disease[22] are being targeted. In addition, DNA vaccines for malaria, AIDS, and many other diseases are in development[23]. DNA vaccines have also been used to prevent allergic response[24].

### Oligonucleotides:

Oligonucleotides are short single-stranded segments of DNA that upon cellular internalization can selectively inhibit the expression of a single protein. For antisense applications, oligonucleotides interact and form a duplex with the mRNA or the pre-mRNA and inhibit their translation or processing, consequently inhibiting protein biosynthesis. For antigene applications, oligonucleotides must enter the cell nucleus; form a triplex with the double- stranded genomic DNA, and inhibits the translation as well as the transcription process of the protein. On the molecular level, numerous mechanisms have been proposed to explain the basis of oligonucleotide action[25–27]. For therapeutic purposes, oligonucleotides can be used to selectively block the expression of proteins that are implicated in diseases[28]. With successful antisense inhibition of proteins in animal models, the first antisense drug, fomivirsen sodium (Vitravene, Isis Pharmaceuticals, Carlsbad, CA) was approved for the treatment of cytomegalovirus retinitis in AIDS patients in 1998[29]. Antisense oligonucleotides such as MG98 and ISIS 5132, designed to inhibit the biosynthesis of DNA methyltransferase and c-raf kinase, respectively, are in human clinical trials for cancer[30]. Synthetic antisense DNA oligonucleotides and oligonucleotide analogs[31], which inhibit the replication of several infectious agents such as hepatitis C virus[32], human cytomegalovirus[33], human immunodeficiency virus and papilloma virus[34–43], have also been designed.

### Aptamers:

DNA - Aptamers are double-stranded nucleic acid segments that can directly interact with proteins[10]. Aptamers interfere with the molecular functions of disease-implicated proteins or those that participate in the transcription or translation processes. Aptamers are preferred over antibodies in protein inhibition owing to their specificity, non-immunogenicity, and stability of pharmaceutical formulation[44]. DNA-aptamers that have demonstrated promise in intervention of pathogenic protein biosynthesis are HIV-1 integrase enzyme[45].

### DNAzymes:

DNAzymes are analogs of ribozymes with greater biological stability[28]. The RNA backbone chemistry is replaced by the DNA motifs that confer improved biological stability. DNAzyme directed against the vascular endothelial growth factor receptor 2 was confirmed to be capable of tumor suppression by blocking angiogenesis upon intratumoral injections in mice[46].

## GENE TRANSFER TECHNOLOGIES

Gene transfer technologies or DNA delivery methods can be classified into 3 general types; electrical techniques, mechanical transfection, and vector assisted delivery systems.

### Mechanical and electrical techniques:

Mechanical and electrical strategies of introducing naked DNA into cells include microinjection, particle bombardment, the use of pressure, and electroporation. Microinjection is highly efficient since one cell at a time is targeted for DNA transfer; however, this precision is achieved at the expense of time. Ballistic transfer of gold micro-particles can be achieved using particle bombardment equipment such as the gene gun. Electroporation uses high-voltage electrical current to facilitate DNA transfer. This technique results in high cell mortality and therefore is not suitable for clinical use[47–50].

### Vector-assisted delivery systems:

Vector-assisted DNA/gene delivery systems can be classified into 2 types based on their origin; biological viral DNA delivery systems and chemical nonviral delivery systems. In viral delivery systems, nonpathogenic attenuated viruses can be used as delivery systems for genes/DNA molecules; especially plasmids[51–53]. These viral DNA-delivery vectors include both RNA and DNA viruses. The viruses used as gene therapy vectors can be classified into 4 types; retroviruses[54], adenoviruses, adeno-associated viruses[55] and Herpes simplex viruses. Gene expression using viral vectors has been achieved with high transfection efficiencies in tissues such as kidney[56], heart muscle[57], eye[55], and ovary[58]. Viruses are currently used in more than 70% of human clinical gene therapy trials world-wide[59]. Gene therapy using viral systems has made considerable progress for the treatment of a wide range of diseases, such as muscular dystrophy[57], AIDS[60], and cancer[61]. The only approved gene therapy treatment (Gendicine) delivers the transgene using a recombinant adenoviral vector[20]. DNA delivery using viral vectors has been extensively reviewed[52,53,62]. The first-generation retroviral vectors were largely derived from oncoretroviruses, such as the Maloney murine leukemia virus (MMuLv), and were unable to transfer genes into non-dividing cells[63,64]. This limited the potential for their application as a delivery system in gene therapy. The utilization of the lentivirus family of retroviruses has overcome this shortcoming. Lentiviruses, which include Human immunodeficiency virus type1 (HIV-1), bovine immunodeficiency virus (BIV), feline immunodeficiency virus (FIV) and simian immunodeficiency virus (SIV), are able to transfer genes to non-dividing cells[64,65].

Retroviral vectors used in gene therapy are replication deficient, such that they are unable to replicate in the host cell and can infect only one cell[66,67]. This characteristic, although essential for the safety of viral vectors in gene therapy, imposes restrictions on the amounts of virus that can safely be administered[68,69]. Retroviral-mediated delivery of therapeutic DNA has been widely used in clinical gene therapy protocols, including the treatment of cancers, such as melanoma[70] and ovarian cancer[71], adenosine deaminase deficiency-severe combined immune deficiency (ADA-SCID)[72,73] and Goucher's disease[74]. Retroviral vectors are capable of transfecting high populations (45-95%) of primary human endothelial and smooth muscle cells, a class of cells that are generally extremely difficult to transfer[75].

Adenoviruses have been used to deliver therapeutic DNA to patients suffering from metastatic breast, ovarian and melanoma cancers[76–78]. Indeed, the severe immune response of the host contributes to the limited survival of the adenoviral DNA in the targeted cells and results in a transient expression of the therapeutic gene since the adenoviral DNA is lost over time[79–83]. First- generation adenoviral vectors were able to accommodate the introduction of therapeutic genes over 7 Kb long (but rarely larger) into targeted cells[84]. However, the generation of gutless adenoviral vectors, which lack all viral genes, has facilitated adenoviral delivery of up to 30 Kb of a therapeutic DNA sequence[85–88] with decreased toxicity[89]. Adenoviral-mediated gene transfer in COS-7 cells was significantly higher than that achieved by liposomal delivery systems[90].

The use of adeno-associated viral (AAV) vectors provides an alternative to adenoviral vectors for gene therapy and a means for long-term gene expression with a reduced risk of adverse reactions upon administration of the vector[91,92]. AAV viruses are linear, single stranded DNA parvoviruses that are not associated with any disease in humans[93]. In humans, the site of AAV viral DNA integration is on chromosome 19[94,95]. In the engineering of AAV vectors, most of the AAV genome can be replaced with the therapeutic gene[96], which significantly reduces potential adverse responses of the host to viral infection. However, the size of the therapeutic gene is limited to approximately 5 Kb[97,98]. First generation adeno-associated viruses had a very small capacity of ~4.7 Kb for encapsulation of the plasmid DNA cargo. Recent reports demonstrate efficient production of second-generation AAV with higher encapsulating capabilities[99]. It has been demonstrated that adenoviruses in formulations may lose their potency after storage in commonly used pharmaceutical vials[100].

Herpes simplex virus (HSV) vector is a large and relatively complex enveloped, double-stranded DNA virus that has the capacity to encode large therapeutic genes and, like AAV, can remain latent in infected cells providing the potential for long term expression of the therapeutic gene[101]. Although, able to infect many cell types, HSV vectors currently are limited in their use by vector toxicity[102].

Non-viral delivery systems have the greatest advantage over viral delivery systems is the lack of immune response and ease of formulation and assembly. Commonly used non-viral vectors for delivery of DNA-based therapeutics can be classified into 3 major types; Naked DNA delivery systems, polymeric delivery systems, and liposomal delivery systems[30,103–105].

Naked DNA can be administered via two possible routes, either by *ex vivo* delivery or by *in vivo* delivery. The *ex vivo* method of naked DNA delivery has been used successfully for the introduction of DNA into endothelial and smooth muscle cells[106,107], its reliance on the culture of harvested cells renders it unsuitable for many cell types. *In vivo* delivery of naked DNA was first described in 1990[108]. Efficiency of the delivery of naked DNA can be improved when administered in a pressure-mediated fashion[107,109]. Particle bombardment technology enables the localized delivery of DNA readily into skin or muscle[110]. Another technique for delivery of naked DNA directly into target cells is electroporation. The successful delivery of DNA by electroporation *in vivo* has been reported in tissues such as skin and muscle[111–114].

In polymeric delivery systems, cationic polymers are used in gene delivery because they can easily complex with the anionic DNA molecules[115]. The mechanism of action of these polycomplexes is based on the generation of a positively charged complex owing to electrostatic interaction of these cationic polymers with anionic DNA[48]. Commonly used polymers include polyethylenimine (PEI)[116], poly-L-lysine (PLL)[117], chitosans[118], and dendrimers[30]. Agents such as folates, transferin, antibodies, or sugars such as galactose and mannose can be incorporated for tissue targeting[30]. Synthetic polymers such as protective interactive non-condensing polymers (PINC), poly-L-lysine, cationic polymers and dendrimers offer an alternative to cationic lipids as a vehicle for DNA delivery into target cells[119–123]. Encapsulation of a DNA molecule or even a therapeutic viral vector within a biodegradable polymer has been demonstrated to permit the controlled release of the DNA in a targeted cell over a period of weeks or months[124,125]. The inclusion of proteins and peptides in the DNA complex that are recognized by receptors on targeted cells has led to an improvement in the efficiency of DNA uptake in several instances[126]. Some polymers have inherent potent pharmacological properties (such as hypercholesterolemia-induced by chitosans) that make them extremely unfavorable for human use[127].

Liposomes are one of the most versatile tools for the delivery of DNA therapeutics[28,103,104,128]. Liposome and drug/lipid complexes have been used for the delivery of the anticancer drugs doxorubicin and daunorubicin[129]. Liposomes can be used as DNA drug delivery systems either by entrapping the DNA-based therapeutics inside the aqueous core or complexing them to the phospholipids lamellae. Liposome can also be used for specialized gene delivery options that include long circulation half-life, sustained and targeted delivery[103].

Numerous studies have demonstrated the use of cationic liposomal formulations for the delivery of different plasmid constructs in a wide range of cells, both *in vivo* and *in vitro*[130]. The use of cationic lipids to transfer DNA into cells was first described as an *in vitro* method of DNA delivery[131]. Cationic liposomes have also been used in clinical trials to deliver therapeutic DNA[132–136]. Cationic liposomal formulations consist of mixtures of cationic and zwitterionic lipids[128,137,138]. Proprietary formulations of cationic lipids such as lipofectamine (Invitrogen, Carlsbad, CA), effectene (Qiagen, Valencia, CA), and tranfectam (Promega, Medison, WI) are commercially available[139], but most of the kits are useful only for *in vitro* experimentation. There are reports of improved efficiency of DNA delivery by cationic lipid via the coupling of specific receptor ligands or peptides to DNA/liposome complexes[126,140–143]. Cytotoxicity of cationic lipids has been established in numerous *in vitro*[144,145] and *in vivo*[146–148] studies. Low transfection efficiencies have been attributed to the heterogeneity and instability of cationic lipoplexes[149]. Another drawback in the use of cationic lipids is their rapid inactivation in the presence of serum[138,150]. Some *in vivo* studies have revealed that the gene transduction responses obtained by cationic lipoisomes were transient and short-lived[151,152].

As an alternative to cationic lipids, the potential of anionic lipids for DNA delivery has been investigated. The safety of anionic lipids has been demonstrated when administered to epithelial lung tissue. In recent years, a few studies, using anionic liposomal DNA delivery vectors have been reported. There have been attempts to incorporate anionic liposomes into polymeric delivery systems. However, these vectors have limited applications, mainly because of (1) inefficient entrapment of DNA molecules within anionic liposomes and (2) lack of toxicity data. Lack of further progress of these systems may be attributed, in part, to the poor association between DNA molecules and anionic lipids, caused by electrostatic repulsion between these negatively charged species[145,146,153–160].

Along with numerous cationic and anionic lipid derivatives, functionalized liposomal formulations serving specific therapeutic objectives have shown promise in gene therapy[103,161,162]. Specialized liposomal delivery platforms include pH-sensitive liposomes, immunoliposomes, and stealth liposomes. pH-Sensitive Liposomes can be generated by the inclusion of 1,2-dioleoyl-3-phosphoethanolamine (DOPE) into liposomes composed of acidic lipids such as cholesterylhemisuccinate or oleic acid. At the neutral cellular pH 7, these lipids have the typical bilayer structure; however, upon endosomal compartmentalization they undergo protonation and collapse into a nonbilayer structure, thereby leading to the disruption and destabilization of the endosomal bilayer, which in turn helps in the rapid release of DNA into the cytoplasm[161]. Efficient gene delivery of the beta-galactosidase and luciferase reporter plasmids has been obtained using pH-sensitive liposomes in a variety of mammalian cell lines[163]. A chemical derivative of DOPE, Citraconyl-DOPE, has been used to deliver DNA-based therapeutics to cancer cells, thereby combining the targeting and the rapid endosome-releasing aspects of specialized liposomal delivery systems[164]. A phosphatidylcholine/glycyrrhizin combination was also successful in pH-sensitive gene delivery in mice[165]. Immunoliposomes are sophisticated gene delivery systems that can be used for cell targeting by the incorporation of functionalized antibodies attached to lipid bilayers[162]. Immunoliposomes containing an antibody fragment against the human transferring receptor were successfully used in targeted delivery of tumor-suppressing genes into tumors *in vivo*[166]. Tissue-specific gene delivery using immunoliposomes has been achieved in the brain[167], embryonic tissue[168], and breast cancer tissue[169]. Stealth liposomes are sterically stabilized liposomal formulations that include polyethylene glycol (PEG)-conjugated lipids[103].

## CURRENT STATUS OF GENE THERAPY RESEARCH

Current gene therapy is experimental and has not proven very successful in clinical trials. Little progress has been made since the first gene therapy clinical trial began in 1990. In 1999, gene therapy suffered a major setback with the death of 18-year-old Jesse Gelsinger. Another major blow came in January 2003, when the FDA placed a temporary halt on all gene therapy trials using retroviral vectors in blood stem cells. FDA took this action after it learned that a second child treated in a French gene therapy trial had developed a leukemia-like condition. Both this child and another who had developed a similar condition in August 2002 had been successfully treated by gene therapy for X-linked severe combined immunodeficiency disease (X-SCID), also known as “bubble baby syndrome”.

FDA's Biological Response Modifiers Advisory Committee (BRMAC) met at the end of February 2003 to discuss possible measures that could allow a number of retroviral gene therapy trials for treatment of life-threatening diseases to proceed with appropriate safeguards. In April of 2003 the FDA eased the ban on gene therapy trials using retroviral vectors in blood stem cells.

## RECENT DEVELOPMENTS IN GENE THERAPY

Nanotechnology and gene therapy yields treatment to torpedo cancer (March, 2009); The School of Pharmacy in London is testing a treatment in mice, which delivers genes wrapped in nanoparticles to cancer cells to target and destroy hard-to-reach cancer cells[170]. Results of world's first gene therapy for inherited blindness show sight improvement (April, 2008); UK researchers from the UCL Institute of Ophthalmology and Moorefield's Eye Hospital NIHR Biomedical Research Centre have announced results from the world's first clinical trial to test a revolutionary gene therapy treatment for a type of inherited blindness. The results, published in the New England Journal of Medicine, show that the experimental treatment is safe and can improve sight. The findings are a landmark for gene therapy technology and could have a significant impact on future treatments for eye disease[171,172].

Previous information on this trial (May 1, 2007); A team of British doctors from Moorfields Eye Hospital and University College in London conduct first human gene therapy trials to treat Leber's congenital amaurosis, a type of inherited childhood blindness caused by a single abnormal gene. The procedure has already been successful at restoring vision for dogs. This is the first trial to use gene therapy in an operation to treat blindness in humans[173].

A combination of two tumor suppressing genes delivered in lipid-based nanoparticles drastically reduces the number and size of human lung cancer tumors in mice during trials conducted in The University of Texas M. D. Anderson Cancer Center and the University of Texas Southwestern Medical Center[174]. Researchers at the National Cancer Institute (NCI), part of the National Institutes of Health, successfully reengineer immune cells, called lymphocytes, to target and attack cancer cells in patients with advanced metastatic melanoma. This is the first time that gene therapy is used to successfully treat cancer in humans[175].

Gene therapy is effectively used to treat two adult patients for a disease affecting nonlymphocytic white blood cells called myeloid cells. Myeloid disorders are common and include a variety of bone marrow failure syndromes, such as acute myeloid leukemia. The study is the first to show that gene therapy can cure diseases of the myeloid system (http://www.cincinnatichildrens.org/March 31, 2006). A research team at the University of California, Los Angeles is able to transport genes into the brain using liposomes coated with polyethylene glycol. The transfer of genes into the brain is a significant achievement because viral vectors are too big to get across the blood-brain barrier. This method has potential for treating Parkinson's disease[176].

RNA interference or gene silencing may be a new way to treat Huntington's. Short pieces of double-stranded RNA (short, interfering RNAs or siRNAs) are used by cells to degrade RNA of a particular sequence. If a siRNA is designed to match the RNA copied from a faulty gene, then the abnormal protein product of that gene will not be produced[177]. New gene therapy approach repairs errors in messenger RNA derived from defective genes. Technique has potential to treat the blood disorder thalassaemia, cystic fibrosis, and some cancers[178]. Gene therapy for treating children with X-SCID (bubble boy) disease is stopped in France when the treatment resulted in leukemia in one of the patients[179]. Researchers at Case Western Reserve University and Copernicus Therapeutics are able to create tiny liposomes 25 nanometers across that can carry therapeutic DNA through pores in the nuclear membrane[180]. Sickle cell is successfully treated in mice[181].
